# Supplementary figures and images for: Comprehensive Proteomic Analysis Revealed a Large Number of Newly Identified Proteins in the Small Extracellular Vesicles of Milk from Late-Stage Lactating Cows
Source: Animals (Basel). 2021 Aug 26;11(9):2506. doi: 10.3390/ani11092506 (PMC8470060; doi:10.3390/ani11092506)

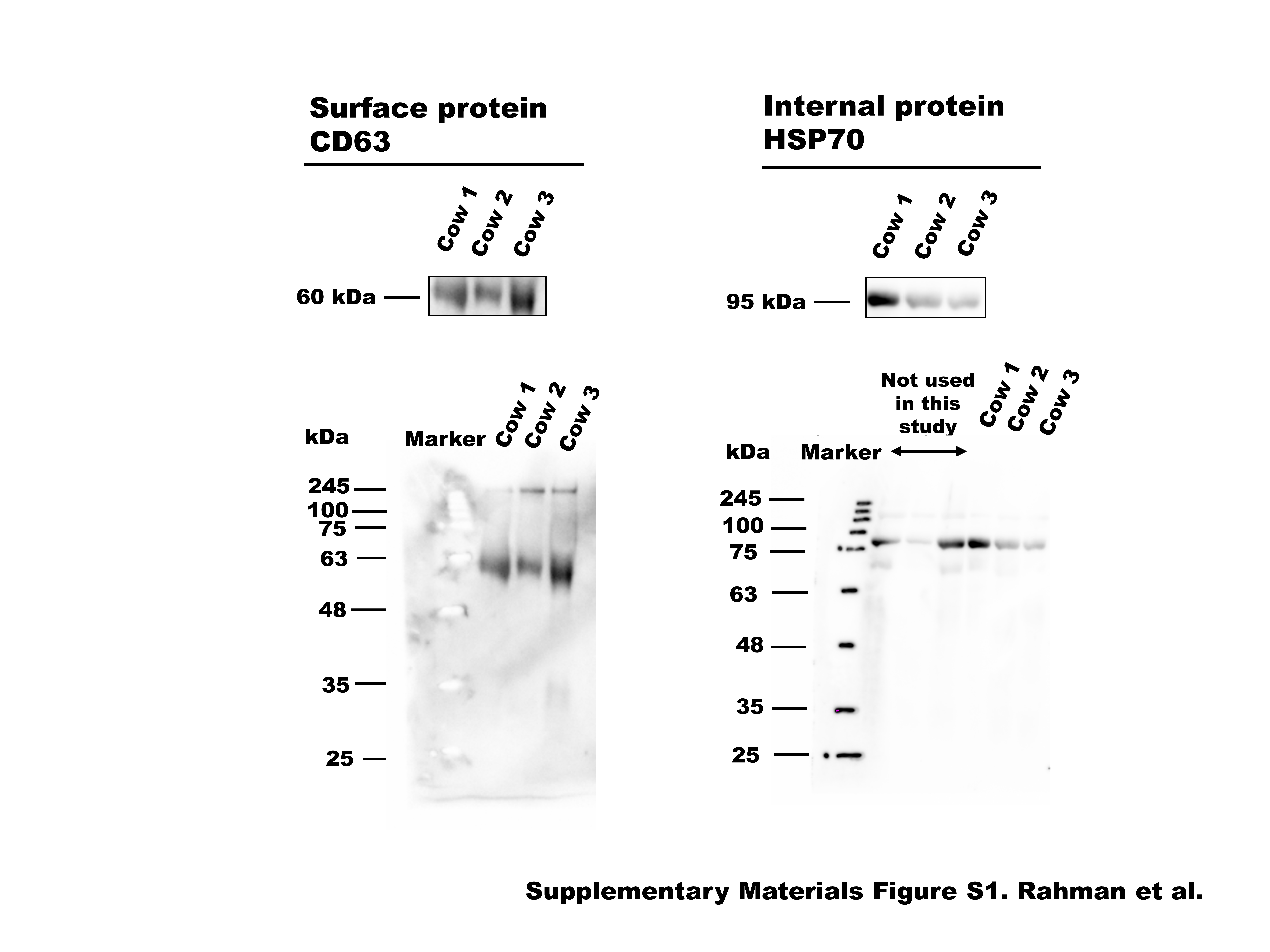

Supplement: Supplementary file 1 [file animals-11-02506-s001.zip › animals-1332722-online supplementary/author proofed supplementary-animals-1332722.png]
